# Dairy Product Consumption Patterns and Associated Sociodemographic and Lifestyle Factors Among Adults from Selected Peruvian Cities

**DOI:** 10.3390/nu18172819

**Published:** 2026-08-28

**Authors:** Youmi Paz-Olivas, Samuel Durán-Agüero, Jacksaint Saintila

**Affiliations:** 1 Área de Investigación, Instituto de Nutrición y Seguridad Alimentaria, Lima 15026, Peru; ypazo2050@gmail.com; 2 Escuela de Nutrición y Dietética, Universidad Femenina Sagrado Corazón (UNIFÉ), La Molina, Lima 15023, Peru; 3 Facultad de Ciencias de la Rehabilitación y Calidad de Vida, Universidad San Sebastian, Providencia 7500000, Chile; samuel.duran@uss.cl; 4 Research Group on Nutritional Psychology and Public Health, Universidad Peruana Unión, Lima 15464, Peru

**Keywords:** dairy products, dietary intake, socioeconomic status, lactose intolerance, adults, food consumption, Peru

## Abstract

**Background:** Dairy products provide protein, calcium, and other essential nutrients, but intake may vary across sociodemographic and lifestyle groups. **Objective:** To characterize dairy consumption patterns and identify factors associated with consuming ≥3 dairy servings/day among adults recruited from selected Peruvian cities. **Methods:** A cross-sectional study was conducted from April to June 2026. Of the 1619 eligible participants, 203 (12.5%) reported no dairy consumption and were analyzed separately for reasons for non-consumption; the final analytical sample included 1265 dairy consumers. Dairy intake was assessed using a culturally adapted questionnaire with content validity evaluated by expert judgment. Descriptive analyses, bivariate tests, and multivariable logistic regression were performed. **Results:** Mean age was 33.0 ± 13.0 years, and 61.2% were women. The most commonly consumed products were cheese (90.9%), yogurt (85.4%), whole milk (71.2%), and *quesillo* or fresh cheese (60.8%). Overall, 16.3% consumed ≥3 dairy servings/day. Participants recruited from cities in the Highlands (aOR = 0.54; 95% CI: 0.37–0.79) and Amazon (aOR = 0.59; 95% CI: 0.40–0.87) had lower odds of reaching this threshold than those from the Coast. Middle socioeconomic status was associated with lower odds (aOR = 0.48; 95% CI: 0.34–0.67), whereas physical activity ≥3 times/week was associated with higher odds (aOR = 1.53; 95% CI: 1.09–2.14). Perceived lactose intolerance was the most frequently reported reason for non-consumption (46.8%). **Conclusions:** Consumption of ≥3 dairy servings/day was uncommon. Socioeconomic differences were the most consistent correlate, while geographic and physical activity associations warrant cautious interpretation.

## 1. Introduction

Milk and dairy products are nutrient-dense foods that provide high-quality protein, calcium, phosphorus, vitamin B12, potassium, and other micronutrients, although their composition varies according to product type and fortification. These foods make an important contribution to the intake of several essential nutrients, particularly calcium and protein [1]. Both nutrients play a key role in maintaining bone structure throughout adulthood and limiting age-related bone loss [2]. For example, a review concluded that dairy consumption as part of a healthy dietary pattern may improve bone mineral density in middle-aged and older adults, although the evidence varies according to the type of dairy product and the population studied [3]. In addition, dairy proteins may contribute to the maintenance of muscle mass and function, particularly among older adults at risk of sarcopenia [4]. Therefore, examining dairy consumption patterns among adults and older adults is warranted.

In Peru, dietary guidance recommends that adults consume 2–3 servings of dairy products per day [5,6]. Dietary guidelines in other settings also commonly recommend approximately three servings or cup-equivalents of dairy products per day, primarily from milk, yogurt, and cheese [7]. However, consuming dairy products, even on a regular basis, does not necessarily mean meeting the recommended intake, as portion sizes and daily consumption frequency may be insufficient. Among Chilean adults, Morales et al. [8] found that only 23.7% met the daily recommendation despite the frequent consumption of cheese, milk, and yogurt. Similarly, a multicenter study [9] conducted among Latin American university students reported that only 28.8% achieved the recommended three daily servings, with substantial variation across countries. In the United States, an analysis of the National Health and Nutrition Examination Survey (NHANES) found that only 13.6% of adults met the recommended intake [10].

Dairy product consumption is not a homogeneous behavior, as it may include fluid milk, yogurt, cheese, fresh cheese (quesillo), kefir, and other fermented products, as well as whole-fat, reduced-fat, or lactose-free varieties [11]. Preferences and consumption levels vary according to the type of product and population characteristics. Importantly, an equivalent number of servings does not necessarily imply equivalent nutritional quality, as dairy products may differ substantially in fat, added sugar, sodium content, fermentation, and degree of processing. Among Chilean adults, cheese, milk, and yogurt consumption differed according to age, geographic region, and socioeconomic status [8], whereas a study conducted across ten European countries within the EPIC cohort found substantial variation in the consumption of these products, highlighting the influence of dietary and cultural contexts [12]. Likewise, sociodemographic and lifestyle factors may be differentially associated with the consumption of milk, cheese, and yogurt [13]. Consequently, both the choice of specific dairy products and adherence to recommended intake may be influenced by commercial availability, culinary traditions, family habits, sensory preferences, and digestive tolerance. These factors are further shaped by structural determinants such as income, education, purchasing power, and physical access to safe and refrigerated foods. A positive gradient between income, education, and dairy consumption has been reported [14], and foods of animal origin tend to represent a greater relative cost for lower-income populations [15]. In addition, limitations in transportation, distribution, and local availability may restrict dairy consumption, particularly in rural or geographically dispersed areas [16]. Finally, dislike of the taste, certain beliefs regarding the health effects of dairy products, and perceived lactose intolerance may contribute to reducing or avoiding dairy consumption, even in the absence of a confirmed clinical diagnosis [17].

Peru is characterized by marked geographic, productive, and sociocultural diversity across its Coast, Highlands, and Amazon regions, which differ in climate, production systems, livelihoods, and population characteristics [18]. Although dairy farming is practiced throughout all three natural regions, important differences exist in production systems, milk processing, and the commercialization of dairy products [19]. These regional characteristics may contribute to territorial differences in the availability, accessibility, and selection of dairy products. However, the available evidence from Peru remains fragmented and has focused primarily on specific populations or settings, such as older adults receiving care in healthcare facilities, among whom inadequate consumption frequencies or intakes of only one to two dairy servings per day have been reported [20,21]. By simultaneously examining product-specific consumption, an intake threshold, associated sociodemographic and lifestyle factors, and reasons for non-consumption, this study provides evidence from an understudied Latin American setting and contributes to understanding dairy consumption across heterogeneous geographic and socioeconomic contexts. Peruvian dietary guidance recommends 2–3 servings of dairy products per day [5]. In the present study, ≥3 servings/day was used as the analytical threshold, corresponding to the upper bound of this recommended range. Therefore, the aim of the present study was to characterize dairy product consumption patterns and determine the factors associated with consuming ≥3 dairy servings/day among adults recruited from selected Peruvian cities. In addition, the main reasons for dairy non-consumption were described.

## 2. Materials and Methods

### 2.1. Study Design, Setting, and Participants

A cross-sectional study was conducted between April and June 2026 among adults recruited from selected locations in Peru. Data collection covered the country’s three natural regions—the Coast, Highlands, and Amazon—and used both face-to-face and online survey modalities. Eligible participants were individuals aged ≥18 years who had resided in Peru for at least five years and voluntarily provided informed consent. Individuals younger than 18 years, those who did not provide informed consent, and participants who were pregnant or breastfeeding were excluded. Duplicate records and records containing implausible or internally inconsistent information required for the analyses were also excluded from the analytical dataset.

The operational sampling framework included nine purposively selected locations to ensure geographic coverage of the three natural regions: Metropolitan Lima–Callao, Trujillo, and Piura on the Coast; Cusco, Huancayo, and Cajamarca in the Highlands; and Iquitos, Pucallpa, and Tarapoto in the Amazon. A non-probability sampling strategy was used. Study locations were selected purposively, whereas participants within each location were recruited by convenience. Recruitment efforts sought to include participants of different sexes and age groups, although no predefined numerical quotas were established.

### 2.2. Recruitment Target and Analytical Sample

A target of approximately 1500 participants was established a priori as an operational recruitment goal, with an intended geographic distribution of approximately 500 participants from each of Peru’s three natural regions. This target was intended to ensure adequate recruitment across the selected study locations and was not based on probability-sampling assumptions or intended to provide population-representative estimates.

A total of 1713 survey records were initially collected. After the initial eligibility screening, 94 records were excluded (83 because of pregnancy or breastfeeding, nine because of invalid informed consent, and two because participants were younger than 18 years), leaving 1619 eligible participants. Of these, 203 reported no dairy consumption and followed the questionnaire’s skip pathway, which collected reasons for non-consumption but not the covariates required for the main analyses; these participants were therefore retained only for the descriptive analysis of non-consumption. Among the 1416 dairy consumers, a further 151 records with duplicate, implausible, internally inconsistent, or non-classifiable information in variables required for the analyses were excluded during data-quality screening. The final complete-case analytical sample comprised 1265 dairy consumers, and this same sample was used for all descriptive, bivariate, and multivariable analyses.

Specifically, the final sample comprise 412 participants from the Coast (Metropol-itan Lima–Callao, *n* = 197; Trujillo, *n* = 120; Piura, *n* = 95), 433 from the Highlands (Cusco, *n* = 166; Huancayo, *n* = 136; Cajamarca, *n* = 131), and 420 from the Amazon (Iquitos, *n* = 150; Pucallpa, *n* = 150; Tarapoto, *n* = 120).

### 2.3. Procedure

Data were collected using the same structured questionnaire in two administration modes: (a) interviewer-administered paper forms and (b) self-administered electronic forms developed in Google Forms. In Metropolitan Lima–Callao, questionnaires were administered face-to-face by trained interviewers. In the remaining study locations, both face-to-face and online administration were used. For the online modality, the survey link was disseminated primarily through social media and instant messaging applications, particularly WhatsApp, with support from local data collection teams. Interviewers received training on questionnaire administration, neutral reading of the questions, clarification of operational queries, and confidentiality procedures.

### 2.4. Variables and Instruments

**Sociodemographic and lifestyle questionnaire.** Sociodemographic information included age, sex, natural region of residence, area of residence, nationality, and educational attainment. Age was analyzed both continuously and in three categories (18–34, 35–59, and ≥60 years). Physical activity was assessed using a single self-reported item asking whether participants engaged in physical activity at least three times per week for more than 30 min per session, with yes/no response options. The questionnaire described such activity as requiring substantial physical effort and explicitly indicated that walking was not included. Usual dietary pattern was assessed through self-classification. Participants selected the category that best described their habitual diet after being provided with brief descriptions of Western, Mediterranean, strict vegetarian/vegan, lacto-ovo vegetarian, and pescatarian patterns, with an additional “other” option. This variable was treated as a self-perceived dietary pattern rather than an objective measure of dietary adherence.

**Approximate socioeconomic status (SES).** Approximate household SES was derived from the educational attainment and main occupation of the household’s primary income earner using an operational adaptation of the Adimark-ESOMAR socioeconomic classification [8,22]. The Adimark-ESOMAR approach combines education and occupation to generate six socioeconomic categories (A, B, Ca, Cb, D, and E). Because the response categories available in the Peruvian questionnaire did not reproduce all educational sublevels of the original classification, participant responses were mapped to the corresponding educational categories using a predefined operational procedure.

Education was classified as follows: no formal education = level 1, primary education = level 2, secondary education = level 4, technical education = level 5, university education = level 6, and postgraduate education = level 7. Occupations were mapped to the six ordered occupational categories of the Adimark-ESOMAR matrix. The resulting categories were collapsed for analysis into high (A/B), middle (Ca/Cb), and low (D/E).

**Body mass index.** Body weight and height were self-reported by participants as approximate values in kilograms and meters, respectively. BMI was calculated as weight in kilograms divided by height in meters squared (kg/m^2^). For descriptive analyses, nutritional status was classified according to age-specific cutoff points established by the Peruvian Ministry of Health. Among adults aged 18–59 years, BMI was categorized as underweight (<18.5 kg/m^2^), normal weight (18.5–24.9 kg/m^2^), overweight (25.0–29.9 kg/m^2^), or obesity (≥30.0 kg/m^2^) [23]. Among adults aged ≥60 years, the corresponding age-specific criteria were applied: underweight (BMI ≤23.0 kg/m^2^), normal weight (>23.0 to <28.0 kg/m^2^), overweight (≥28.0 to <32.0 kg/m^2^), and obesity (≥32.0 kg/m^2^) [24]. In the multivariable regression analyses, BMI was modeled as a continuous variable per 1 kg/m^2^ increase.

**Dairy product consumption questionnaire.** Dairy consumption was assessed using a culturally adapted Peruvian version of a questionnaire previously validated in Chile [8,25]. The instrument identified dairy consumers and non-consumers and assessed habitual consumption of cheese, quesillo or fresh cheese, yogurt, homemade yogurt, kefir, whole, semi-skimmed, skimmed, lactose-free and raw milk, as well as reasons for non-consumption. The questions assessed usual or habitual consumption frequency rather than intake over a fixed retrospective recall period. Total dairy intake was obtained from a direct question on usual total dairy consumption frequency and converted to weekly equivalents. Response categories were converted to weekly serving equivalents as follows: no consumption = 0; once every 15 days = 0.5; once/week = 1; 2–3 times/week = 2.5; 4–5 times/week = 4.5; ½ serving/day = 3.5; 1 serving/day = 7; 2 servings/day = 14; 3 servings/day = 21; 4 servings/day = 28; and ≥5 servings/day = 35 servings/week. Product-specific consumption frequencies were not summed to define total dairy intake. Standard serving sizes were 200 mL for milk, 125 g for yogurt, 30 g for cheese or fresh cheese, and 50 g for *quesillo* [8]. The primary outcome was defined as consumption of ≥3 servings/day (≥21 servings/week), corresponding to the upper limit of the 2–3 servings/day recommended for Peruvian adults by the National Institute of Health/CENAN [5,6]. In the original study, the questionnaire underwent content validation through expert judgment using Lawshe’s method, with a content validity ratio (CVR) cutoff of ≥0.78. The Peruvian adaptation was subsequently evaluated by five registered dietitian nutritionists for relevance, clarity, and appropriateness. The content validity of the dairy consumption section was considered adequate (Aiken’s V = 0.87).

### 2.5. Ethical Considerations

The study was approved by the Ethics Committee of Universidad Peruana Unión (2026-CEUPeU-010; 9 April 2026). All participants provided written or electronic informed consent before enrollment. All study procedures were conducted in accordance with the Declaration of Helsinki, and no directly identifiable personal information was collected.

### 2.6. Statistical Analysis

Data were analyzed using R version 4.4.1 (R Foundation for Statistical Computing, Vienna, Austria). Categorical variables were summarized as frequencies and percentages, and continuous variables as means and standard deviations. Comparisons between groups were performed using Pearson’s chi-square test for categorical variables and Welch’s *t*-test for continuous variables.

All primary analyses were conducted in the complete-case analytical sample of 1265 dairy consumers. No missing data were imputed. Dairy product consumption prevalence was calculated for each product, and participant characteristics were compared according to consumption of <3 or ≥3 dairy servings/day. Crude associations were estimated using separate binary logistic regression models for each predictor. A multivariable logistic regression model was then fitted including age group, sex, natural region, area of residence, nationality, approximate socioeconomic status, physical activity, BMI, and self-perceived dietary pattern. BMI was modeled continuously per 1 kg/m^2^ increase, and all covariates were entered simultaneously based on conceptual relevance rather than statistical significance in the bivariate analyses. Participants reporting strict vegetarian/vegan and lacto-ovo vegetarian dietary patterns were combined because of the small number in the former category. Results are presented as crude and adjusted odds ratios (ORs) with 95% confidence intervals (CIs).

Sensitivity analyses included replacing approximate socioeconomic status with participant educational level while retaining the same covariates and analytical sample (Table S1), and further adjusting the main model for survey administration mode (face-to-face vs. online). A restricted model was also fitted among participants from cities in which both administration modes were represented (Table S2). Multicollinearity was assessed using the variance inflation factor (VIF). All tests were two-sided, and *p* < 0.05 was considered statistically significant. Reasons for dairy non-consumption were analyzed descriptively among the 203 participants who reported not consuming dairy products.

## 3. Results

Table 1 summarizes the sociodemographic, anthropometric, and lifestyle characteristics of the 1265 dairy consumers included in the final analytical sample, stratified by sex. The mean age was 33.0 ± 13.0 years, 61.2% were women, and most participants lived in urban areas (87.1%). Significant differences by sex were observed for natural region of residence, nationality, educational level, physical activity, mean BMI, and BMI classification. In contrast, age, area of residence, approximate socioeconomic status, and self-perceived dietary pattern did not differ significantly by sex. Overall, 16.3% of participants consumed ≥3 dairy servings/day, with no significant difference between women and men.

**Figure 1:**
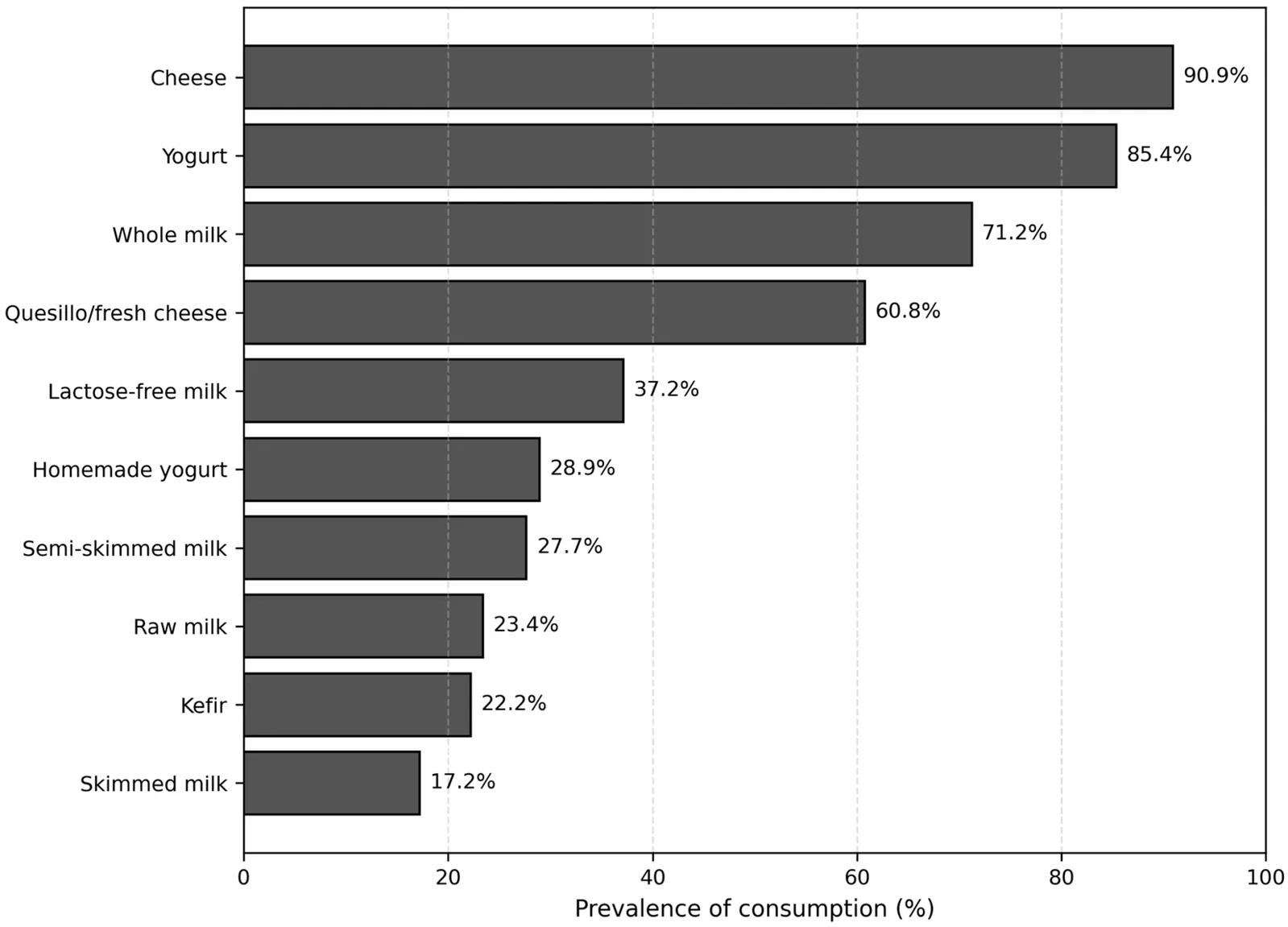
Prevalence of dairy product consumption among dairy consumers. **Note.** Bars represent the percentage of participants who reported habitual consumption of each dairy product among consumers of dairy products. Whole milk, semi-skimmed milk, and lactose-free milk categories include both canned and UHT presentations. Values are expressed as percentages.

Figure 1 shows that cheese was the most commonly consumed dairy product (90.9%), followed by yogurt (85.4%), whole milk (71.2%), and *quesillo* or fresh cheese (60.8%). Consumption of the remaining dairy products was substantially less frequent.

**Figure 2:**
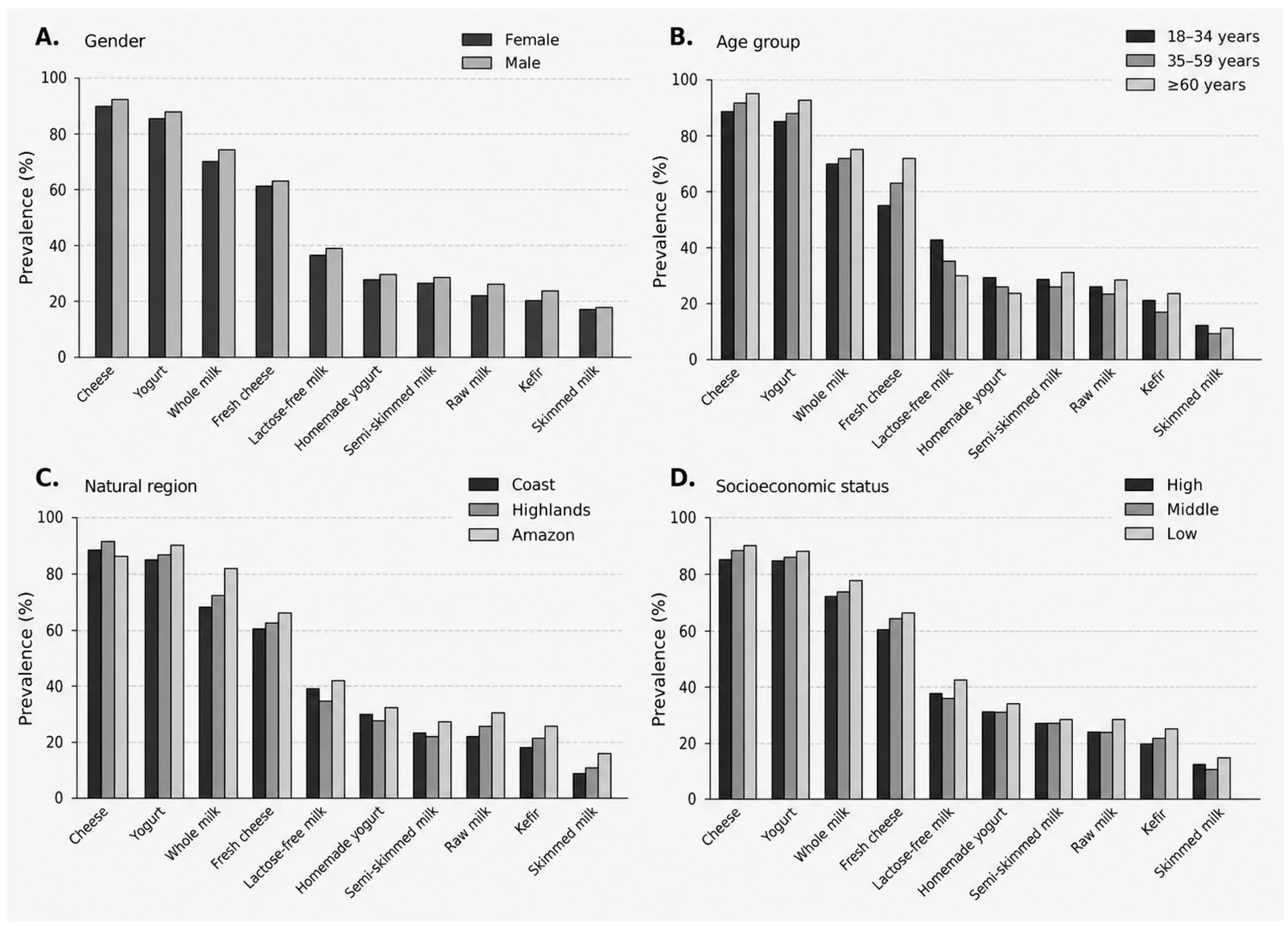
Prevalence of dairy product consumption according to sociodemographic characteristics. **Note.** Bars represent the percentage of dairy consumers who reported habitual consumption of each dairy product, stratified by (**A**) gender, (**B**) age group, (**C**) natural region of residence, and (**D**) socioeconomic status. Whole milk, semi-skimmed milk, and lactose-free milk categories include both canned and UHT presentations.

Figure 2 shows the distribution of dairy product consumption according to sex, age group, natural region, and approximate socioeconomic status. Cheese and yogurt remained the most commonly consumed dairy products across all subgroups. Descriptive variation was more apparent by age and natural region, particularly for whole milk, *quesillo* or fresh cheese, lactose-free milk, raw milk, and kefir, whereas consumption patterns were generally similar between women and men.

Table 2 compares participant characteristics according to consumption of <3 versus ≥3 dairy servings/day. Participants consuming ≥3 servings/day were younger than those consuming <3 servings/day (30.4 ± 11.3 vs. 33.6 ± 13.2 years; *p* < 0.001). The distribution also differed significantly by age group, natural region, educational level, approximate socioeconomic status, and physical activity. Participants reaching the ≥3-servings/day threshold were more frequently from the Coast (44.2%), classified in the high socioeconomic group (38.3%), and physically active at least three times per week (64.1%). No significant differences were observed for sex, area of residence, nationality, BMI, BMI classification, or self-perceived dietary pattern.

Table 3 presents the crude associations with consumption of ≥3 dairy servings/day. Increasing age was associated with lower odds of reaching the threshold (OR = 0.98 per year; 95% CI: 0.97–0.99), and participants aged ≥60 years had lower odds than those aged 18–34 years (OR = 0.38; 95% CI: 0.15–0.97). Lower odds were also observed among participants from the Highlands (OR = 0.53; 95% CI: 0.37–0.77) and Amazon (OR = 0.57; 95% CI: 0.39–0.81), those with middle or low approximate socioeconomic status, and those reporting a Western dietary pattern. Conversely, physical activity ≥3 times/week was associated with higher odds of consuming ≥3 dairy servings/day (OR = 1.76; 95% CI: 1.29–2.40).

Table 4 presents the multivariable logistic regression model for consumption of ≥3 dairy servings/day. After adjustment, participants from the Highlands (aOR = 0.54; 95% CI: 0.37–0.79) and Amazon (aOR = 0.59; 95% CI: 0.40–0.87) had lower odds of reaching the ≥3-servings/day threshold than those from the Coast. Middle socioeconomic status was also associated with lower odds compared with high socioeconomic status (aOR = 0.48; 95% CI: 0.34–0.67), whereas physical activity ≥3 times/week was associated with higher odds (aOR = 1.53; 95% CI: 1.09–2.14). No significant associations were observed for sex, area of residence, nationality, low socioeconomic status, BMI, or self-perceived dietary pattern. No evidence of problematic multicollinearity was detected (maximum VIF = 1.47).

**Figure 3:**
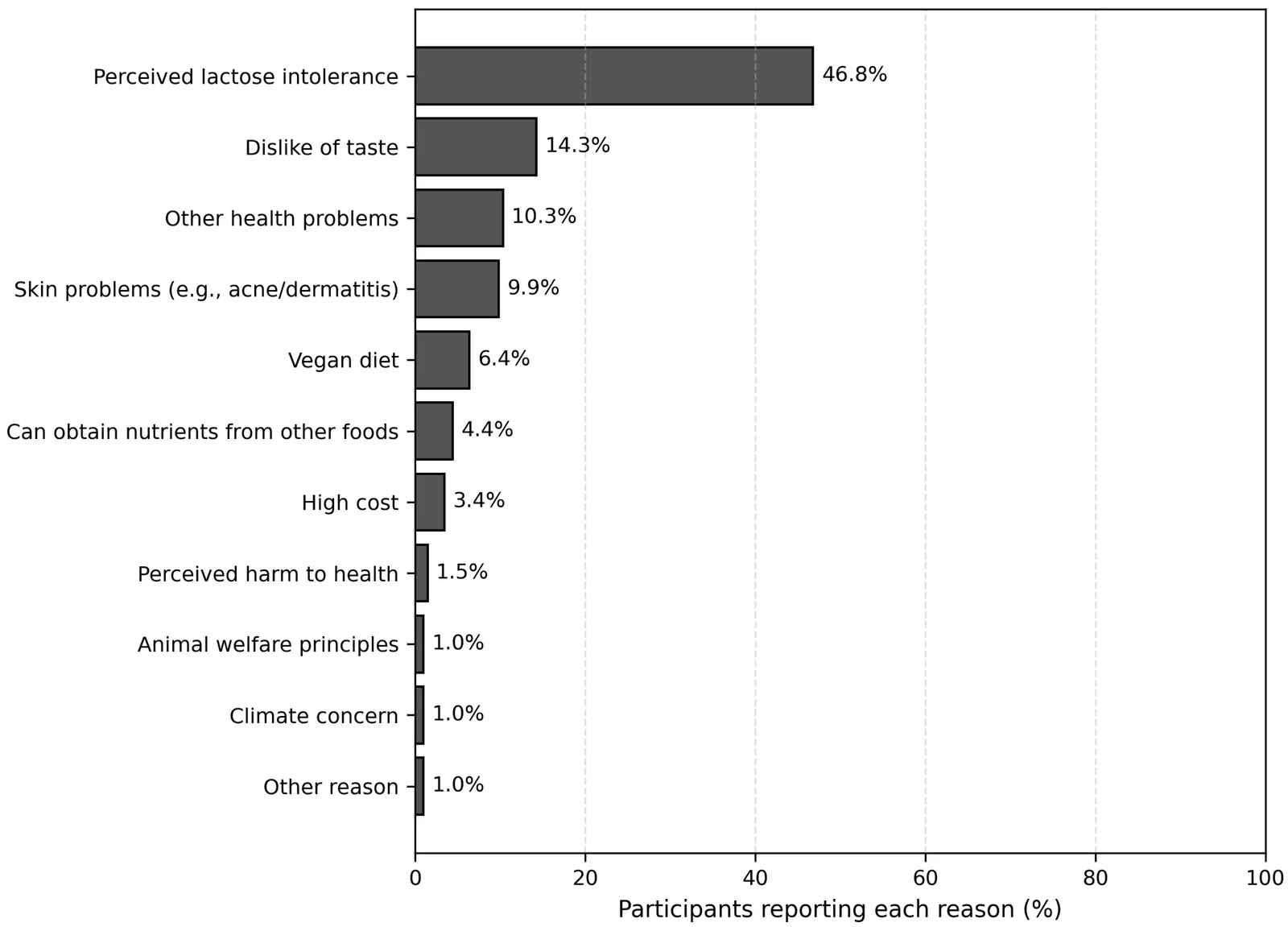
Reasons for non-consumption of dairy products. **Note.** Bars represent the percentage of the 203 participants who reported not consuming dairy products and selected each reason for non-consumption. Lactose intolerance reflects participants’ self-perception and was not clinically confirmed.

Figure 3 presents the reasons reported for dairy non-consumption among the 203 non-consumers. Perceived lactose intolerance was the most frequently reported reason (46.8%), followed by dislike of taste (14.3%), other health problems (10.3%), and skin-related concerns such as acne or dermatitis (9.9%).

In the sensitivity analysis, when approximate socioeconomic status was replaced by participant educational level, the inverse associations for the Highlands and Amazon and the positive association with physical activity remained significant (Table S1). Further adjustment for survey administration mode in the full analytical sample produced minimal changes in the main estimates, and survey mode itself was not associated with the outcome (Table S2). However, when the analysis was restricted to cities in which both survey modes were represented (N = 1068), the associations with natural region and physical activity were attenuated and no longer statistically significant, whereas the inverse association with middle socioeconomic status remained (Table S2).

## 4. Discussion

The present study evaluated dairy product consumption patterns and factors associated with consuming ≥3 dairy servings/day among adults recruited from selected cities across Peru’s three natural regions. The main findings indicate that, although consumption of at least one type of dairy product was common, the proportion consuming ≥3 servings/day was low. The most commonly consumed products were cheese, yogurt, whole milk, and *quesillo* or fresh cheese. In the adjusted model, participants recruited from cities in the Highlands or Amazon and those with middle socioeconomic status had lower odds of consuming ≥3 servings/day, whereas self-reported physical activity ≥3 times/week was associated with higher odds. The association with middle socioeconomic status was the most consistent across sensitivity analyses, while the regional and physical activity associations were attenuated in the analysis restricted to cities in which both survey administration modes were represented. In addition, among participants who did not consume dairy products, the most frequently reported reason was perceived lactose intolerance.

One of the main findings was the low proportion of participants consuming ≥3 dairy servings/day. Only 16.3% of dairy consumers reached this intake threshold. This finding is consistent with that reported in Chile by Morales et al. [8], who found that only 23.7% of adults met the dairy intake recommendation. Similarly, a multicenter study among Latin American university students showed that, although 66% consumed at least one serving of dairy products per day, failure to meet the recommendation of ≥3 servings/day remained common, particularly in countries with a lower Human Development Index [9]. This trend has also been reported outside Latin America. For example, analyses based on NHANES indicated that nearly all US adults consumed less than the recommended 2.5–3 daily servings [26], and more recent data showed that only a minority of the population met the dairy intake recommendation [27]. The low prevalence of consumption at the ≥3-servings/day threshold may be explained by the fact that habitual dairy consumption does not necessarily translate into consuming three servings per day. In addition, factors such as cost, availability, perceived lactose intolerance, and local dietary practices may limit the regular inclusion of dairy products in the daily diet.

Another important finding was the high prevalence of cheese, yogurt, and whole milk consumption. In our study, cheese was the most commonly consumed dairy product, followed by yogurt, whole milk, and *quesillo* or fresh cheese. In Chile, cheese was also identified as the most commonly consumed dairy product, followed by milk and yogurt [8]. In the Peruvian context, this finding is also consistent with information from the Ministry of Agrarian Development and Irrigation, according to which seven out of ten Peruvians consume cheese, making it one of the most widely accepted dairy products nationwide [28]. A trend similar to our findings has also been observed in the United States, where fluid milk consumption has progressively declined and cheese consumption has increased over recent decades [29]. However, this finding differs from that of another study, in which milk accounted for the largest proportion of total dairy intake, followed by yogurt and other fermented products, and then cheese [12]. Overall, the findings of the present study suggest that efforts to increase dairy intake should consider the range of dairy products habitually consumed rather than focusing exclusively on milk, including widely consumed products such as cheese, yogurt, and *quesillo* or fresh cheese.

Differences according to the natural region in which participants were recruited were also observed. In the primary adjusted model, participants recruited from cities in the Highlands and Amazon had lower odds of consuming ≥3 dairy servings/day than those recruited from cities on the Coast. These associations persisted when participant educational level replaced socioeconomic status in the sensitivity model. However, they were attenuated and no longer statistically significant when the analysis was restricted to cities in which both face-to-face and online survey modes were represented. Therefore, the regional estimates should be interpreted cautiously and should not be considered evidence of population-level differences among Peru’s natural regions. Evidence from Chile has also documented geographic variation in dairy consumption [8]. Differences in food availability, affordability, distribution systems, local dietary practices, and characteristics of the selected cities may contribute to the observed pattern. Indeed, intensive production systems and specialized dairy cattle in Peru are concentrated mainly on the Coast, whereas extensive, less technologically developed systems with infrastructure limitations predominate in the Highlands and Amazon [30]. Nevertheless, Cajamarca and Arequipa are among the country’s leading dairy-producing regions [19], indicating that greater regional production does not necessarily ensure adequate individual consumption. Accordingly, dairy production and individual consumption should not be assumed to follow the same geographic pattern, and further studies using probability-based sampling are needed to clarify these territorial differences.

Regarding socioeconomic status, participants in the middle socioeconomic group had lower odds of consuming ≥3 dairy servings/day than those in the high socioeconomic group, whereas the estimate for the low socioeconomic group did not reach statistical significance after adjustment. Notably, the inverse association for middle socioeconomic status remained when the analysis was restricted to cities with both survey administration modes, suggesting greater stability of this finding. Another study similarly reported that individuals with low or middle socioeconomic status had lower odds of meeting the recommendation than those with high socioeconomic status [6]. Our findings are also consistent with those of Velhinho et al. [14], who observed a positive gradient between income, education, and dairy product consumption. One possible explanation is that households with fewer economic resources face greater constraints in regularly purchasing animal-source foods; indeed, these foods are relatively more expensive in lower-income settings [15]. In addition, household income has been associated with greater household availability of low-fat milk [31]. However, this relationship is not uniform across all products. For example, Morales et al. [8] found higher consumption of certain dairy beverages among participants with low socioeconomic status, possibly because of specific food assistance programs. In the present study, socioeconomic status was an approximate household-level indicator derived from an adapted Adimark-ESOMAR classification; consequently, the association should be interpreted as a socioeconomic gradient within the study sample rather than as a direct measure of household income or purchasing power.

Another relevant finding was that participants who reported physical activity ≥3 times per week for >30 min per session had higher odds of consuming ≥3 servings of dairy products per day in the primary adjusted model. This result is consistent with the multicenter study by Gajardo et al. [9], in which low dairy consumption was associated with lower levels of physical activity and other unfavorable health indicators. It is also consistent with a study conducted among healthy Spanish adults, which found that those who exercised more frequently consumed greater amounts of milk and dairy products, along with other components of a healthy lifestyle [32]. Similarly, Lu et al. [33] reported associations between dairy consumption and physical activity among young Chinese women, although the magnitude and direction of these associations varied according to the dairy product evaluated. Physically active individuals may be more likely to adhere to nutritional recommendations [34]. In addition, exercise may encourage the selection of protein- and calcium-rich foods to support muscle recovery and bone health [35].

Among the 203 participants who did not consume dairy products, the most frequently reported reason was perceived lactose intolerance (46.8%). This finding is consistent with the Chilean study by Morales et al. [8], in which lactose intolerance was also the main barrier to dairy consumption. However, in our study, this condition was self-reported and was not confirmed through diagnostic testing; therefore, it should be interpreted as perceived intolerance rather than a clinical diagnosis. This distinction is particularly important because agreement among self-reported symptoms, objectively measured lactose malabsorption, and response to dietary restriction is variable [36]. Moreover, perceived intolerance may lead to the unnecessary exclusion of dairy products; indeed, individuals who consider themselves lactose intolerant consume fewer dairy products and less calcium [37]. Many people with lactose intolerance can also tolerate small amounts of lactose [38]. Therefore, professional guidance should prioritize appropriate assessment and, when indicated, recommend lactose-free products, low-lactose cheeses, or fermented yogurts, while avoiding unnecessary restrictions that could compromise calcium and other nutrient intakes.

### 4.1. Public Health Implications

Public health and nutrition-promotion strategies should emphasize practical guidance on appropriate dairy portions and consider differences in affordability, availability, and local food preferences when promoting dairy consumption. Where dairy intake is limited or not feasible, guidance should include nutritionally appropriate alternatives to support adequate calcium and protein intake. In addition, the high frequency of perceived lactose intolerance highlights the importance of professional assessment and education regarding individual lactose tolerance, lactose-free products, low-lactose cheeses, and fermented dairy foods, thereby reducing unnecessary dietary restrictions.

### 4.2. Strengths, Limitations, and Future Considerations

The main strengths of this study include its relatively large sample, the inclusion of participants from selected cities across Peru’s three natural regions, the use of a culturally adapted dairy consumption questionnaire with content validation, and the evaluation of multiple dairy products. In addition, the use of a common analytical sample and sensitivity analyses strengthened the consistency of the statistical approach.

Several limitations should be considered. The cross-sectional design precludes causal inference, and the non-probability sampling strategy limits generalizability to the Peruvian population. The analytical sample was predominantly urban and relatively highly educated, while adults aged ≥60 years represented only a small proportion; therefore, the observed dairy consumption estimates may not reflect those of the general Peruvian adult population. Regional findings should also be interpreted cautiously because study locations were purposively selected and the associations were attenuated when analyses were restricted to cities with both survey administration modes. Dairy consumption, physical activity, weight, and height were self-reported, and physical activity was assessed using a single binary item. Dairy intake may therefore be affected by recall and social-desirability bias, with potential misclassification around the ≥3-servings/day threshold. Although the Peruvian adaptation underwent expert-based content evaluation, test–retest reliability and criterion or convergent validity were not established; therefore, content validity should not be interpreted as evidence of full measurement validity. Dietary pattern was based on self-classification, lactose intolerance was perceived rather than clinically confirmed, and socioeconomic status was an approximate measure derived from an adapted Adimark-ESOMAR approach.

Future studies should use probability-based sampling, validated dietary and physical activity measures, and more detailed dietary assessment methods. Longitudinal studies are also needed to clarify the temporal relationships observed and to determine whether geographic and socioeconomic differences persist after accounting for contextual and city-level factors.

## 5. Conclusions

In this cross-sectional study, the proportion of adults recruited from selected Peruvian cities who consumed ≥3 dairy servings/day was low. Although cheese, yogurt, whole milk, and *quesillo* or fresh cheese were frequently consumed, most participants consumed below this threshold. Middle socioeconomic status was consistently associated with lower odds of consuming ≥3 dairy servings/day, whereas associations with natural region and physical activity were less consistent across sensitivity analyses. Among non-consumers, the main reported barrier was perceived lactose intolerance, which was self-reported and not clinically confirmed. These findings highlight socioeconomic differences in dairy intake and the importance of considering perceived lactose intolerance when developing context-appropriate nutrition-promotion strategies.

## Figures and Tables

**Table 1:** Sociodemographic, Anthropometric, and Lifestyle Characteristics by Sex.

Variable	Total (N = 1265)	Female (*n* = 774)	Male (*n* = 491)	*p*-Value
**Age, years, mean ± SD**	33.0 ± 13.0	33.0 ± 12.9	33.2 ± 13.2	0.770
**Age group, *n* (%)**				0.441
18–34	814 (64.3)	499 (64.5)	315 (64.2)	
35–59	386 (30.5)	240 (31.0)	146 (29.7)	
≥60	65 (5.1)	35 (4.5)	30 (6.1)	
**Natural region, *n* (%)**				<0.001
Coast	412 (32.6)	270 (34.9)	142 (28.9)	
Highlands	433 (34.2)	282 (36.4)	151 (30.8)	
Amazon	420 (33.2)	222 (28.7)	198 (40.3)	
**Area of residence, *n* (%)**				0.364
Urban	1102 (87.1)	669 (86.4)	433 (88.2)	
Rural	163 (12.9)	105 (13.6)	58 (11.8)	
**Nationality, *n* (%)**				0.005
Peruvian	1225 (96.8)	741 (95.7)	484 (98.6)	
Other nationality	40 (3.2)	33 (4.3)	7 (1.4)	
**Educational level, *n* (%)**				0.005
No formal/primary	35 (2.8)	29 (3.7)	6 (1.2)	
Secondary	218 (17.2)	135 (17.4)	83 (16.9)	
Technical	268 (21.2)	159 (20.5)	109 (22.2)	
University	641 (50.7)	401 (51.8)	240 (48.9)	
Postgraduate	103 (8.1)	50 (6.5)	53 (10.8)	
**Socioeconomic status, *n* (%)**				0.195
High	329 (26.0)	195 (25.2)	134 (27.3)	
Middle	790 (62.5)	480 (62.0)	310 (63.1)	
Low	146 (11.5)	99 (12.8)	47 (9.6)	
**Physical activity ≥3 times/week, *n* (%)**				<0.001
No	600 (47.4)	431 (55.7)	169 (34.4)	
Yes	665 (52.6)	343 (44.3)	322 (65.6)	
**BMI, kg/m^2^, mean ± SD**	25.9 ± 4.1	25.5 ± 4.0	26.4 ± 4.3	<0.001
**BMI classification, *n* (%)**				0.026
Underweight	26 (2.1)	16 (2.1)	10 (2.0)	
Normal	577 (45.6)	378 (48.8)	199 (40.5)	
Overweight	485 (38.3)	283 (36.6)	202 (41.1)	
Obesity	177 (14.0)	97 (12.5)	80 (16.3)	
**Self-perceived dietary pattern, *n* (%)**				0.400
Mediterranean	521 (41.2)	323 (41.7)	198 (40.3)	
Western	271 (21.4)	156 (20.2)	115 (23.4)	
Pescatarian	332 (26.2)	200 (25.8)	132 (26.9)	
Vegetarian/lacto-ovo vegetarian	113 (8.9)	76 (9.8)	37 (7.5)	
Other	28 (2.2)	19 (2.5)	9 (1.8)	
**Meets ≥3 servings/day, *n* (%)**				0.158
No	1059 (83.7)	657 (84.9)	402 (81.9)	
Yes	206 (16.3)	117 (15.1)	89 (18.1)	

**Note.** Data are presented as mean ± standard deviation (SD) or *n* (%), as appropriate. *p*-values were obtained using Welch’s *t*-test for continuous variables and the chi-square test or Fisher’s exact test for categorical variables, as appropriate. BMI, body mass index.

**Table 2:** Characteristics of Dairy Consumers According to the ≥3 Servings/Day Dairy Intake Threshold.

Variable	Total (N = 1265)	<3 Servings/Day (*n* = 1059)	≥3 Servings/Day (*n* = 206)	*p*-Value
**Age, years, mean ± SD**	33.0 ± 13.0	33.6 ± 13.2	30.4 ± 11.3	<0.001
**Age group, *n* (%)**				0.042
18–34	814 (64.3)	668 (63.1)	146 (70.9)	
35–59	386 (30.5)	331 (31.3)	55 (26.7)	
≥60	65 (5.1)	60 (5.7)	5 (2.4)	
**Sex, *n* (%)**				0.158
Female	774 (61.2)	657 (62.0)	117 (56.8)	
Male	491 (38.8)	402 (38.0)	89 (43.2)	
**Natural region, *n* (%)**				<0.001
Coast	412 (32.6)	321 (30.3)	91 (44.2)	
Highlands	433 (34.2)	376 (35.5)	57 (27.7)	
Amazon	420 (33.2)	362 (34.2)	58 (28.2)	
**Area of residence, *n* (%)**				0.086
Urban	1102 (87.1)	915 (86.4)	187 (90.8)	
Rural	163 (12.9)	144 (13.6)	19 (9.2)	
**Nationality, *n* (%)**				0.510
Peruvian	1225 (96.8)	1024 (96.7)	201 (97.6)	
Other nationality	40 (3.2)	35 (3.3)	5 (2.4)	
**Educational level, *n* (%)**				0.011
No formal/primary	35 (2.8)	34 (3.2)	1 (0.5)	
Secondary	218 (17.2)	188 (17.8)	30 (14.6)	
Technical	268 (21.2)	233 (22.0)	35 (17.0)	
University	641 (50.7)	525 (49.6)	116 (56.3)	
Postgraduate	103 (8.1)	79 (7.5)	24 (11.7)	
**Socioeconomic status, *n* (%)**				<0.001
High	329 (26.0)	250 (23.6)	79 (38.3)	
Middle	790 (62.5)	685 (64.7)	105 (51.0)	
Low	146 (11.5)	124 (11.7)	22 (10.7)	
**Physical activity ≥3 times/week, *n* (%)**				<0.001
No	600 (47.4)	526 (49.7)	74 (35.9)	
Yes	665 (52.6)	533 (50.3)	132 (64.1)	
**BMI, kg/m^2^, mean ± SD**	25.9 ± 4.1	25.8 ± 4.0	26.0 ± 4.9	0.627
**BMI classification, *n* (%)**				0.237
Underweight	26 (2.1)	19 (1.8)	7 (3.4)	
Normal	577 (45.6)	483 (45.6)	94 (45.6)	
Overweight	485 (38.3)	414 (39.1)	71 (34.5)	
Obesity	177 (14.0)	143 (13.5)	34 (16.5)	
**Self-perceived dietary pattern, *n* (%)**				0.104
Mediterranean	521 (41.2)	429 (40.5)	92 (44.7)	
Western	271 (21.4)	239 (22.6)	32 (15.5)	
Pescatarian	332 (26.2)	280 (26.4)	52 (25.2)	
Vegetarian/lacto-ovo vegetarian	113 (8.9)	88 (8.3)	25 (12.1)	
Other	28 (2.2)	23 (2.2)	5 (2.4)	

**Note.** Data are presented as mean ± standard deviation (SD) or *n* (%), as appropriate. *p*-values were obtained using Welch’s *t*-test for continuous variables and the chi-square test or Fisher’s exact test for categorical variables, as appropriate. BMI, body mass index.

**Table 3:** Crude Logistic Regression Models for the ≥3 Servings/Day Dairy Intake Threshold.

Variable	Category	*n*	Meets ≥3 Servings/Day, *n* (%)	Crude OR	95% CI	*p*-Value
Age	Per 1-year increase	1265	206 (16.3)	0.98	0.97–0.99	0.002
Age group	18–34	814	146 (17.9)	Ref.		
	35–59	386	55 (14.2)	0.76	0.54–1.06	0.111
	≥60	65	5 (7.7)	0.38	0.15–0.97	0.042
Sex	Female	774	117 (15.1)	Ref.		
	Male	491	89 (18.1)	1.24	0.92–1.68	0.158
Natural region	Coast	412	91 (22.1)	Ref.		
	Highlands	433	57 (13.2)	0.53	0.37–0.77	<0.001
	Amazon	420	58 (13.8)	0.57	0.39–0.81	0.002
Area of residence	Urban	1102	187 (17.0)	Ref.		
	Rural	163	19 (11.7)	0.65	0.39–1.07	0.089
Nationality	Peruvian	1225	201 (16.4)	Ref.		
	Other nationality	40	5 (12.5)	0.73	0.28–1.88	0.512
Educational level	University	641	116 (18.1)	Ref.		
	Postgraduate	103	24 (23.3)	1.37	0.83–2.26	0.211
	Technical	268	35 (13.1)	0.68	0.45–1.02	0.064
	Secondary	218	30 (13.8)	0.72	0.47–1.12	0.142
	No formal/primary	35	1 (2.9)	0.13	0.02–0.98	0.048
Socioeconomic status	High	329	79 (24.0)	Ref.		
	Middle	790	105 (13.3)	0.49	0.35–0.67	<0.001
	Low	146	22 (15.1)	0.56	0.33–0.94	0.029
Physical activity ≥3 times/week	No	600	74 (12.3)	Ref.		
	Yes	665	132 (19.8)	1.76	1.29–2.40	<0.001
BMI	Per 1 kg/m^2^ increase	1265	206 (16.3)	1.01	0.97–1.05	0.574
BMI classification	Normal	577	94 (16.3)	Ref.		
	Underweight	26	7 (26.9)	1.89	0.77–4.63	0.162
	Overweight	485	71 (14.6)	0.88	0.63–1.23	0.459
	Obesity	177	34 (19.2)	1.22	0.79–1.89	0.366
Self-perceived dietary pattern	Mediterranean	521	92 (17.7)	Ref.		
	Western	271	32 (11.8)	0.62	0.41–0.96	0.033
	Pescatarian	332	52 (15.7)	0.87	0.60–1.26	0.448
	Vegetarian/lacto-ovo vegetarian	113	25 (22.1)	1.32	0.81–2.18	0.268
	Other	28	5 (17.9)	1.01	0.38–2.74	0.979

**Note.** OR, odds ratio; CI, confidence interval; BMI, body mass index; Ref., reference category. The outcome was consumption of ≥3 dairy servings/day (1 = yes; 0 = <3 servings/day). Each crude OR was estimated from a separate binary logistic regression model. BMI was modeled continuously per 1 kg/m^2^ increase.

**Table 4:** Adjusted Logistic Regression Analysis of Factors Associated with Consuming ≥3 Dairy Servings per Day.

Variable	Category	*n*	Meets ≥3 Servings/Day, *n* (%)	Adjusted OR	95% CI	*p*-Value
Age group	18–34	814	146 (17.9)	Ref.		
	35–59	386	55 (14.2)	0.77	0.54–1.11	0.158
	≥60	65	5 (7.7)	0.37	0.14–0.96	0.040
Sex	Female	774	117 (15.1)	Ref.		
	Male	491	89 (18.1)	1.20	0.87–1.65	0.276
Natural region	Coast	412	91 (22.1)	Ref.		
	Highlands	433	57 (13.2)	0.54	0.37–0.79	0.002
	Amazon	420	58 (13.8)	0.59	0.40–0.87	0.007
Area of residence	Urban	1102	187 (17.0)	Ref.		
	Rural	163	19 (11.7)	0.87	0.51–1.48	0.616
Nationality	Peruvian	1225	201 (16.4)	Ref.		
	Other nationality	40	5 (12.5)	0.98	0.36–2.66	0.971
Socioeconomic status	High	329	79 (24.0)	Ref.		
	Middle	790	105 (13.3)	0.48	0.34–0.67	<0.001
	Low	146	22 (15.1)	0.61	0.36–1.05	0.075
Physical activity ≥3 times/week	No	600	74 (12.3)	Ref.		
	Yes	665	132 (19.8)	1.53	1.09–2.14	0.014
BMI	Per 1 kg/m^2^ increase	1265	206 (16.3)	1.03	0.99–1.07	0.209
Self-perceived dietary pattern	Mediterranean	521	92 (17.7)	Ref.		
	Western	271	32 (11.8)	0.71	0.44–1.13	0.148
	Pescatarian	332	52 (15.7)	0.82	0.56–1.20	0.299
	Vegetarian/lacto-ovo vegetarian	113	25 (22.1)	1.34	0.80–2.24	0.269
	Other	28	5 (17.9)	0.94	0.34–2.62	0.912

**Note**. aOR, adjusted odds ratio; CI, confidence interval; BMI, body mass index; Ref., reference category. The multivariable model included age group, sex, natural region, area of residence, nationality, approximate socioeconomic status, physical activity, BMI, and self-perceived dietary pattern, all entered simultaneously. Maximum VIF = 1.47, indicating no relevant multicollinearity.

## Data Availability

The anonymized data supporting the findings of this study are available from the corresponding author upon reasonable request, subject to the conditions established by the approving ethics committee.

## Supplementary Materials

The following supporting information can be downloaded at: https://www.mdpi.com/article/10.3390/nu18172819/s1, Table S1: Sensitivity analysis of the adjusted logistic regression model for consuming ≥3 dairy servings per day, replacing socioeconomic status with participant educational level. Table S2: Sensitivity analyses accounting for survey administration mode.
