# Supplementary material for: Dairy Product Consumption Patterns and Associated Sociodemographic and Lifestyle Factors Among Adults from Selected Peruvian Cities
Source: Nutrients. 2026 Aug 28;18(17):2819. doi: 10.3390/nu18172819 (PMC13567231; doi:10.3390/nu18172819)
Supplement: Supplementary file 1 [file nutrients-18-02819-s001.zip › nutrients-4511239-supplementary.pdf]

Supplementary Table S1. Sensitivity analysis of the adjusted logistic regression model for consuming ≥3 dairy servings per day, replacing socioeconomic status with participant educational level

Variable	Category	n	≥3 servings/day, n (%)	aOR	95% CI	p-value
**Age group**	18–34	814	146 (17.9)	Ref.		
	35–59	386	55 (14.2)	0.80	0.55–1.16	0.243
	≥60	65	5 (7.7)	0.43	0.16–1.13	0.088
**Sex**	Female	774	117 (15.1)	Ref.		
	Male	491	89 (18.1)	1.15	0.84–1.59	0.385
**Natural region**	Coast	412	91 (22.1)	Ref.		
	Highlands	433	57 (13.2)	0.57	0.39–0.83	0.004
	Amazon	420	58 (13.8)	0.62	0.42–0.90	0.013
**Area of residence**	Urban	1102	187 (17.0)	Ref.		
	Rural	163	19 (11.7)	0.85	0.50–1.44	0.537
**Nationality**	Peruvian	1225	201 (16.4)	Ref.		
	Other nationality	40	5 (12.5)	0.91	0.34–2.47	0.860
**Participant educational level**	University	641	116 (18.1)	Ref.		
	Postgraduate	103	24 (23.3)	1.53	0.90–2.61	0.117
	Technical	268	35 (13.1)	0.75	0.49–1.14	0.183
	Secondary	218	30 (13.8)	0.83	0.53–1.31	0.422
	No formal/primary	35	1 (2.9)	0.26	0.03–1.99	0.195
**Physical activity ≥3 times/week**	No	600	74 (12.3)	Ref.		
	Yes	665	132 (19.8)	1.54	1.10–2.15	0.011
**BMI**	Per 1 kg/m² increase	1265	206 (16.3)	1.02	0.98–1.06	0.255

**Self-perceived dietary pattern**	Mediterranean	521	92 (17.7)	Ref.		
	Western	271	32 (11.8)	0.72	0.45–1.15	0.170
	Pescatarian	332	52 (15.7)	0.83	0.57–1.22	0.351
	Vegetarian/lacto-ovo vegetarian	113	25 (22.1)	1.29	0.77–2.16	0.325
	Other	28	5 (17.9)	1.04	0.38–2.88	0.932

Note: aOR, adjusted odds ratio; CI, confidence interval; BMI, body mass index; Ref., reference category. The outcome was consumption of ≥3 dairy servings/day (1 = yes; 0 = <3 servings/day). This sensitivity model used the same analytical sample as the main adjusted model (N = 1,265) and retained the same covariates—age group, sex, natural region, area of residence, nationality, physical activity, BMI, and self-perceived dietary pattern—while replacing approximate socioeconomic status with participant educational level. All covariates were entered simultaneously. Participants reporting vegetarian/vegan and lacto-ovo vegetarian dietary patterns were combined because of the small number in the former category.

Supplementary Table S2. Sensitivity analyses accounting for survey administration mode

		Sensitivity model 1: Full analytical sample (N = 1,265)					Sensitivity model 2: Cities with both survey modes (N = 1,068)				
**Variable**	**Category**	**n**	**≥3 servings/day, n (%)**	**aOR**	**95% CI**	**p-value**	**n**	**≥3 servings/day, n (%)**	**aOR**	**95% CI**	**p-value**
**Age group**	18–34	814	146 (17.9)	Ref.			666	103 (15.5)	Ref.		
	35–59	386	55 (14.2)	0.77	0.54–1.11	0.162	351	41 (11.7)	0.73	0.48–1.10	0.135
	≥60	65	5 (7.7)	0.37	0.14–0.98	0.046	51	4 (7.8)	0.60	0.20–1.78	0.361
**Sex**	Female	774	117 (15.1)	Ref.			638	79 (12.4)	Ref.		
	Male	491	89 (18.1)	1.20	0.87–1.66	0.271	430	69 (16.0)	1.27	0.88–1.85	0.201
**Natural region**	Coast	412	91 (22.1)	Ref.			215	33 (15.3)	Ref.		
	Highlands	433	57 (13.2)	0.53	0.36–0.78	0.001	433	57 (13.2)	0.84	0.52–1.37	0.489
	Amazon	420	58 (13.8)	0.58	0.39–0.85	0.006	420	58 (13.8)	0.91	0.56–1.47	0.688
**Area of residence**	Urban	1102	187 (17.0)	Ref.			907	130 (14.3)	Ref.		
	Rural	163	19 (11.7)	0.87	0.51–1.47	0.600	161	18 (11.2)	0.95	0.54–1.65	0.845
**Nationality**	Peruvian	1225	201 (16.4)	Ref.			1029	144 (14.0)	Ref.		
	Other nationality	40	5 (12.5)	0.95	0.35–2.59	0.925	39	4 (10.3)	0.72	0.24–2.16	0.555
**Approximate socioeconomic status**	High	329	79 (24.0)	Ref.			276	60 (21.7)	Ref.		
	Middle	790	105 (13.3)	0.49	0.35–0.68	<0.001	667	73 (10.9)	0.48	0.32–0.70	<0.001
	Low	146	22 (15.1)	0.63	0.36–1.09	0.097	125	15 (12.0)	0.62	0.32–1.18	0.145
**Physical activity ≥3 times/week**	No	600	74 (12.3)	Ref.			513	55 (10.7)	Ref.		
	Yes	665	132 (19.8)	1.51	1.08–2.12	0.017	555	93 (16.8)	1.34	0.90–1.99	0.150
**BMI**	Per 1 kg/m² increase	1265	206 (16.3)	1.02	0.99–1.07	0.212	1068	148 (13.9)	1.01	0.97–1.06	0.599

**Self-perceived dietary pattern**	Mediterranean	521	92 (17.7)	Ref.			409	54 (13.2)	Ref.		
	Western	271	32 (11.8)	0.69	0.43–1.12	0.132	255	30 (11.8)	0.85	0.51–1.43	0.542
	Pescatarian	332	52 (15.7)	0.80	0.55–1.18	0.268	296	41 (13.9)	0.95	0.60–1.49	0.826
	Vegetarian/lacto-ovo vegetarian	113	25 (22.1)	1.32	0.79–2.22	0.291	84	19 (22.6)	1.87	1.01–3.46	0.045
	Other	28	5 (17.9)	0.94	0.34–2.61	0.906	24	4 (16.7)	1.17	0.37–3.66	0.789
**Survey administration mode**	Face-to-face	658	104 (15.8)	Ref.			461	46 (10.0)	Ref.		
	Online	607	102 (16.8)	1.10	0.79–1.54	0.555	607	102 (16.8)	1.53	1.03–2.27	0.037

Note. aOR, adjusted odds ratio; CI, confidence interval; BMI, body mass index; Ref., reference category. The outcome was consumption of ≥3 dairy servings/day (1 = yes; 0 = <3 servings/day). Sensitivity model 1 included the full complete-case analytical sample and added survey administration mode (face-to-face vs. online) to the main adjusted model. Sensitivity model 2 was restricted to participants from Trujillo, Piura, Cusco, Huancayo, Cajamarca, Iquitos, Pucallpa, and Tarapoto, where both survey modes were represented; Metropolitan Lima–Callao was excluded because all observations there were face-to-face. Both models included age group, sex, natural region, area of residence, nationality, approximate socioeconomic status, physical activity, BMI, self-perceived dietary pattern, and survey administration mode. BMI was modeled continuously per 1 kg/m² increase. Strict vegetarian/vegan and lacto-ovo vegetarian categories were combined because of the small number in the former category. All covariates were entered simultaneously.
